# Supplementary material for: Reference Gene Selection for Expression Analyses by qRT-PCR in Dendroctonus valens
Source: Insects. 2020 May 27;11(6):328. doi: 10.3390/insects11060328 (PMC7349367; doi:10.3390/insects11060328)
Supplement: Supplementary file 1 [file insects-11-00328-s001.pdf]

	Min	Opt	Max
Primer Size	18	20	22
Primer Tm	57	60	63
Primer GC%	40	50	60
Product Size	150	200	250

Figure S1 The design strategy of Primer3Plus

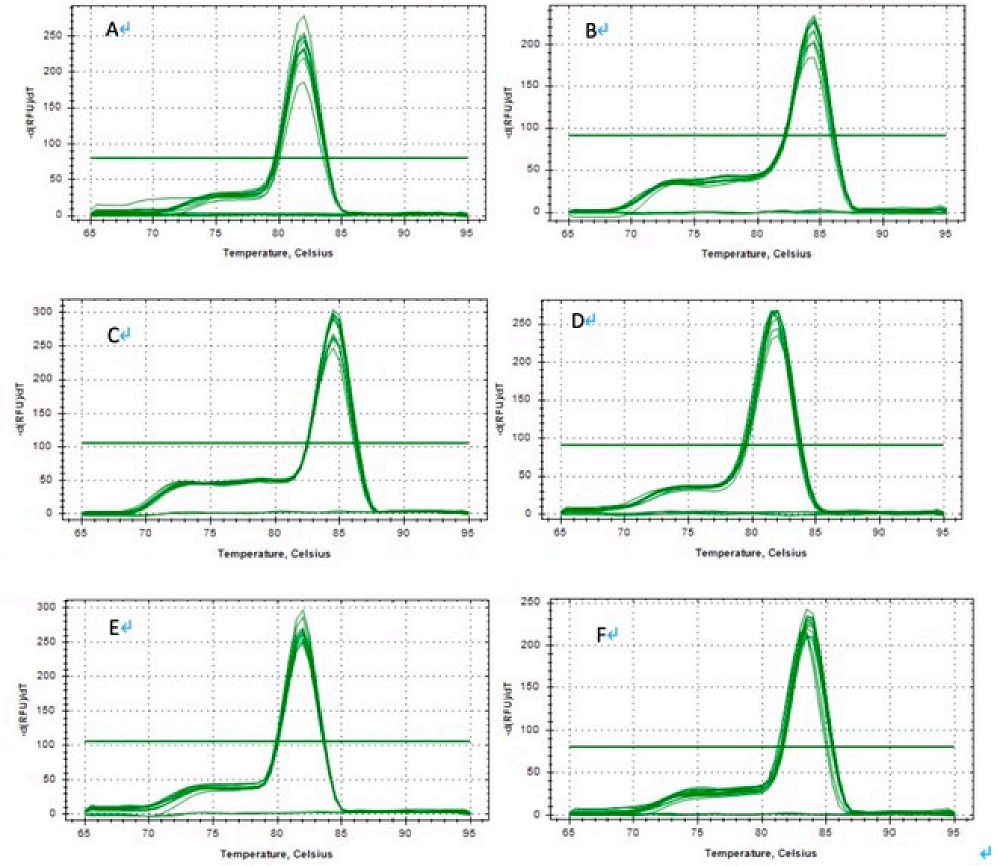

Figure S2 Specificity of real-time qRT-PCR amplification in *Dendroctonus valens*. Melting curves of six genes [*ACT*（A）, *SDHA*（B）, *18S rRNA*（C）, *RPS18*（D）, *CYP4G55*（E）and *TUB*（F）] reveal single peaks.
